# Biomaterials for Reproductive Restoration: Translating Engineering Innovations

**DOI:** 10.34133/bmr.0307

**Published:** 2026-01-22

**Authors:** Chungmo Yang, Hyuk Sang Yoo

**Affiliations:** ^1^ Institute for Molecular Science and Fusion Technology, Kangwon National University, Chuncheon 24341, Republic of Korea.; ^2^ Department of Biomedical Materials Engineering, Kangwon National University, Chuncheon 24341, Republic of Korea.; ^3^ Kangwon Radiation Convergence Research Center, Kangwon National University, Chuncheon 24341, Republic of Korea.; ^4^ Institute of Biomedical Science, Kangwon National University, Chuncheon 24341, Republic of Korea.

## Abstract

Advances in cancer therapy, delayed parenthood, and an increasing number of reproductive disorders have intensified the need for the effective preservation of fertility. However, current clinical strategies such as ovarian tissue cryopreservation, transplantation, and hormonal stimulation remain limited in scope and efficacy. Biomaterials have emerged as powerful tools to overcome these limitations, enabling fertility preservation and functional restoration of reproductive and endocrine systems. Recent progress has included the development of hydrogel-based systems for in vitro follicle maturation, bioengineered scaffolds for ovarian tissue support, and artificial ovaries capable of hormone secretion and oocyte development. These platforms are increasingly incorporating immunomodulatory features to address rejection and enhance graft integration. Beyond preservation, biomaterials are also being harnessed to repair reproductive damage caused by conditions such as primary ovarian insufficiency, intrauterine adhesions, and endometriosis. Through tunable biochemical and mechanical properties, materials can direct tissue regeneration, modulate inflammation, and restore physiological functions. Emerging technologies, including biofabrication with reproductive-specific bioinks, organoid models, hormone-responsive systems, and artificial intelligence-driven biomaterial designs, are accelerating innovation toward translational applications. Collectively, these developments represent a paradigm shift in reproductive medicine from passive preservation to active regenerative strategies. This review highlights the state-of-the-art biomaterial-enabled fertility restoration and outlines future directions toward personalized, functional, and clinically viable solutions.

## Introduction

Fertility preservation has become a pressing issue in reproductive medicine, especially given evolving demographic and clinical landscapes. Recent advances in cancer treatment have improved survival rates, yet nearly 1 million reproductive-age women receive cancer diagnoses annually, many of whom undergo gonadotoxic therapies [1–4]. Concurrently, sociocultural changes, including delayed childbearing, heighten the risk of age-related fertility decline. Global fertility rates in 2023 averaged 2.3 children per woman—a notable decrease from 4.9 in the 1950s (World Bank data). Projections indicate that by 2025, several regions will experience accelerated declines, with Taiwan (1.11), South Korea (1.12), Singapore (1.17), Ukraine (1.22), and Hong Kong (1.24) recording nearly one child per woman [5–8]. These alarmingly low fertility rates threaten national stability by reducing the workforce, straining economies, and creating challenges in maintaining the population, prompting governments to implement social and economic measures to promote childbirth.

Despite these clinical and societal challenges, prevailing fertility preservation options remain inadequate. Embryo and oocyte cryopreservation require ovarian stimulation, making them unsuitable for urgent cases or hormone-sensitive cancers [9]. Ovarian tissue cryopreservation, while applicable to prepubertal patients, yields success owing to post-transplantation ischemic injury and limited follicle survival [10,11]. These limitations underscore the need for innovative strategies to preserve and restore reproductive functions. Accordingly, investigating the ovarian microenvironment and developing bioengineered regenerative solutions are essential for meeting the requirements of patient populations.

Biomaterials provide an unprecedented opportunity to overcome the limitations of current fertility preservation and restoration strategies by enabling the design of biomimetic environments that support folliculogenesis, tissue integration, and long-term endocrine function [12,13]. Unlike cryopreservation matrices, engineered biomaterials—such as hydrogels, decellularized extracellular matrices (dECMs), and nanofibrous scaffolds—deliver bioactive cues dynamically interacting with reproductive cells and tissues [14]. Hydrogels composed of alginate, polyethylene glycol (PEG), collagen, and gelatin support 3-dimensional (3D) follicle culture, allowing oocyte maturation in vitro under physiologically relevant conditions. Similarly, injectable dECM-based platforms have demonstrated angiogenic potential and immune compatibility in ovarian tissue transplantation (OTT) models, thereby reducing ischemic loss and enhancing graft survival [15]. Stem cell-laden constructs that mimic the ovarian niche have demonstrated promise in restoring hormonal function in preclinical models [16,17]. These systems function not only as passive carriers but also as interactive platforms for cellular communication, mechanotransduction, and sustained biofactor delivery. The programmable nature of these materials permits tuning of mechanical stiffness, degradation rates, and cytokine release, thereby enabling precise control over the reproductive microenvironment.

Biomaterial-based reproductive engineering represents a shift from supportive to functional systems, yielding clinically marked outcomes for patients with gonadotoxic insults or tissue dysfunction. The application of biomaterials in reproductive medicine extends beyond fertility preservation to treating reproductive disorders. Pathologies such as primary ovarian insufficiency (POI), polycystic ovary syndrome (PCOS), intrauterine adhesion (IUA), Asherman’s syndrome, endometriosis, and uterine factor infertility (UFI) substantially contribute to female reproductive dysfunction, often resulting in irreversible tissue damage [18,19]. Conventional treatments—hormone replacement therapy (HRT), surgical adhesion removal, and ovarian drilling—fail to restore the native structure, microenvironment, or function of reproductive tissues [20,21]. By contrast, biomaterial-based strategies offer regenerative solutions through controlled immunomodulation, targeted cell recruitment, and localized delivery of bioactive factors. For example, hydrogel scaffolds embedded with growth factors—vascular endothelial growth factor (VEGF) and growth differentiation factor 9 (GDF-9)—have demonstrated promise in restoring ovarian vasculature and promoting follicle development in POI models [22,23]. Likewise, ECM-based uterine patches have enabled endometrial regeneration in Asherman’s syndrome, whereas 3D-engineered constructs incorporating anti-inflammatory agents have effectively reduced fibrosis in endometriosis models [24,25]. These approaches aim to rebuild rather than bypass damaged tissues. Moreover, combining biomaterials with stem cells or synthetic biology tools permits the design of responsive, self-regulating systems that align with endogenous hormonal cycles. Given that many reproductive disorders involve mechanical, inflammatory, and hormonal factors, the integrative capacity of biomaterials represents a powerful tool for multifaceted interventions.

This review synthesizes current advances and future directions at the intersection of biomaterials and reproductive medicine, with an emphasis on their application in fertility preservation and reproductive tissue regeneration. After outlining the limitations of existing fertility preservation strategies, engineered solutions such as follicle culture, OTT, and hormone-producing artificial ovaries have been developed. Furthermore, the review examines the use of biomaterials to treat major reproductive disorders beyond preservation, addressing both tissue-level damage and endocrine dysfunction. It highlights enabling technologies such as 3D bioprinting and organoids and discusses translational challenges, including vascularization, immunogenicity, and regulatory hurdles. This review aimed to provide a comprehensive framework for developing next-generation reproductive platforms that deliver personalized, functionally robust fertility solutions in the clinic.

## Biomaterial-Based Fertility Preservation, Current and Beyond

Over the past 2 decades, significant clinical and technological advancements have addressed the shortcomings of traditional fertility preservation approaches [26]. Orthotopic transplantation of cryopreserved ovarian tissue has produced over 200 live births worldwide, illustrating its potential to restore both endocrine and reproductive functions [27]. Moreover, in vitro maturation (IVM) of oocytes has emerged as a viable option for patients unable to undergo hormonal stimulation [28,29]. Promising emerging protocols involving follicle activation, encapsulation, and culture have been demonstrated for generating mature oocytes in vitro [30]. However, these developments remained inconsistent and dependent on patient-specific variables such as age, ovarian reserve, and disease context. Key challenges include ischemic damage following transplantation, incomplete folliculogenesis in vitro, and a lack of essential microenvironmental cues for follicle survival and maturation [31,32]. Furthermore, long-term endocrine restoration—crucial for quality of life post-treatment—has rarely been sustained by current methods [33]. Regulatory hurdles, costs, and limited scalability further constrain clinical translation at scale. These limitations underscore the necessity for a paradigm shift from conventional cryopreservation toward strategies that replicate native reproductive tissue structure and function. Therefore, integrating the principles of tissue engineering and regenerative medicine has emerged as a key frontier in reproductive science.

The exploration of biomaterials for fertility preservation arose from limitations in conventional approaches that emphasize storage over active restoration of ovarian function. Incorporating biomaterials responds to the ovarian microenvironment’s inherent complexity, which current methods fail to replicate [34,35]. The ovary is dynamic, undergoing continuous remodeling, orchestrated hormonal feedback, and spatially defined interactions between follicles and the surrounding stroma [36]. Existing clinical strategies, such as oocyte/embryo cryopreservation and OTT, remain passive, emphasizing storage and survival rather than dynamic functionality. Therefore, these approaches frequently fail to attain long-term endocrine restoration, folliculogenesis, or the formation of developmentally competent oocytes post-thaw or post-implantation.

Biomaterials offer a distinctive avenue to bridge this gap by creating tissue-inspired platforms that replicate the physical architecture, biochemical signaling, and mechanical dynamics essential for sustaining ovarian function. Scientifically, hydrogels and scaffolds can be tailored to emulate the viscoelasticity of ovarian stroma, present ECM-derived ligands, and regulate nutrient and oxygen diffusion—parameters critical for follicle survival and growth [37]. Furthermore, materials can be engineered to respond to hormonal stimuli, degrade synchronously with tissue regeneration, or release growth factors temporally, thereby enabling functional integration surpassing that of conventional cryopreservation or transplantation [38]. Based on these principles, biomaterial systems have transitioned toward experimental therapies. Preclinical studies demonstrated that hydrogel-encapsulated follicles preserve cell viability and produce estradiol, while ECM-derived scaffolds can mitigate ischemic damage following ovarian tissue implantation [39–41]. Early-stage translational research reported endocrine recovery in patients with premature ovarian insufficiency (POI) using autologous tissue embedded in proregenerative matrices, suggesting that biomaterial-based support may extend graft function. Nevertheless, significant challenges persist [42,43]. Although in vitro follicle culture using engineered matrices is advancing, complete oocyte maturation and fertilization remain elusive. Additionally, clinical implementation faces difficulties; the process is labor-intensive and costly, and developing a clinically robust, standardized protocol with stringent quality control constitutes a major barrier for in vitro fertilization (IVF) laboratories.

**Fig. 1.**
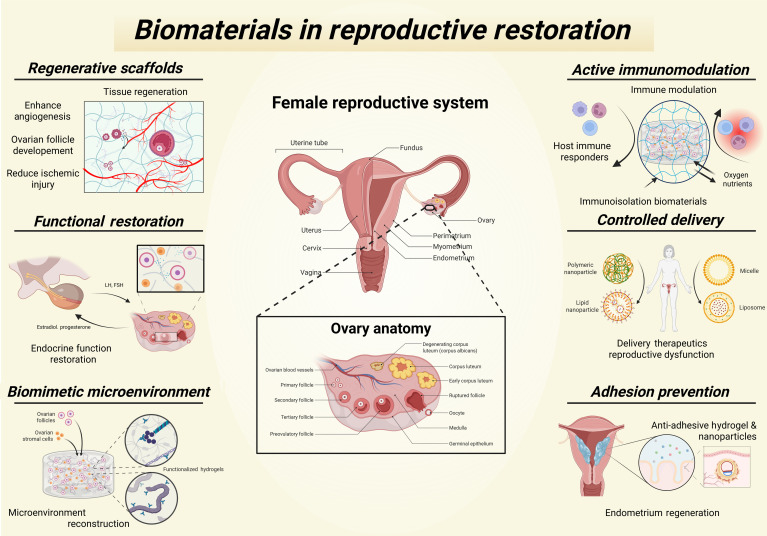
Overview of biomaterial-based strategies for reproductive restoration. A graphical summary of the applications of biomaterials in restoring female reproductive function. The illustration shows the anatomy of the female reproductive system, with a detailed inset of the ovarian structure and folliculogenesis and key therapeutic strategies.

Biomaterial-based strategies are valuable for patients for whom conventional treatments prove insufficient. Prepubertal girls, individuals with hormone-sensitive malignancies, and patients with poor ovarian reserves cannot undergo ovarian stimulation and risk malignant cell reintroduction following tissue transplantation [44–46]. Biomaterials offer a safer, more adaptable approach to culturing isolated follicles, modulating the immune microenvironment, and localizing regenerative cues. Despite these promising results, translation into widespread clinical practice remains limited. Major barriers include variability in material composition, immunogenicity—especially for xenogeneic extracellular matrix (ECM)—and regulatory concerns regarding long-term implantation [47,48]. Manufacturing reproducibility and quality control remain essential for clinical-scale deployment. Nonetheless, both the scientific rationale and clinical need for biomaterial-based fertility preservation continue gaining support. These systems extend beyond preservation to functional restoration, offering endocrine support, follicular maturation, and reproductive autonomy to populations previously deemed ineligible. As the field matures, integration of materials science, developmental biology, and clinical strategies becomes critical for realizing the full translational potential of these engineered reproductive platforms (Fig. 1).

## Engineering Functional Biomaterials for Reproductive Tissues

Biomaterials facilitate engineering of reproductive microenvironments that actively support follicle development, tissue integration, and hormonal functions, thereby transforming fertility preservation into a regenerative strategy (Table 1).

**Table 1.** Summary of biomaterials for reproduction-related applications

Application	Core material	Type	Key effects	Reference
Follicle/oocyte	Chitosan	Hydrogel	Maintained 3D follicular architecture and function for in vitro maturation.	[52–54]
	Alginate	Hydrogel		
	Collagen	Hydrogel		
	Alginate/fibrin	Hydrogel	Improved the rate of meiotically competent oocyte production	[55]
	Collagen/alginate	Hydrogel	Mimicked the ovarian stiffness gradient to enhance folliculogenesis and induce in vitro ovulation.	[62]
	Alginate + Adipose-derived stem cells (ADSCs)	Hydrogel	Improved follicle survival, growth, and antrum formation.	[17]
Ovary	Fibrin + VEGF	Hydrogel	For OTT: Promoted angiogenesis, doubled primordial follicle survival, and restored fertility.	[75]
	Fibrin + mPEG-PLGA nanoparticles	Hydrogel + Nanoparticles	For OTT: Enhanced neovascularization around transplanted ovaries to improve follicle survival.	[76]
	Fibrin + Platelet-rich plasma	Hydrogel	For OTT: Enhances avascular OTT by preserving follicle quality, promoting neovascularization, reducing ischemic damage, and improving functional reproductive outcomes.	[78]
	Dual-layer PEG	Hydrogel capsule	For allogeneic OTT: Supported ovarian tissue survival and restored endocrine function for over 60 d in immunocompetent mice.	[60]
	Dual-layer PEG	Hydrogel capsule	For allogeneic OTT: Precluded immune rejection by preventing donor-specific antibody formation and T cell infiltration.	[79]
	PEG	Hydrogel	For hormonal restoration: Used a synthetic hydrogel to support artificial ovarian tissue and re-establish physiological, cyclic hormone secretion.	[45]
	Multilayer alginate capsules	Hydrogel/capsule	For hormonal restoration (artificial ovary): Encapsulated ovarian cells to restore cyclic hormone secretion and correct uterine atrophy.	[85]
		Hydrogel/capsule	For hormonal restoration: Corrected uterine atrophy and other abnormalities of ovarian failure via in vivo transplantation of the engineered construct.	[86]
	Alginate	Hydrogel microbeads	For hormonal restoration (artificial ovary): Enabled minimally invasive delivery, restored endocrine function, and avoided mammary gland hyperplasia.	[88]
	Spheroids + Gelatin	Hydrogel/spheroid complex	For hormonal restoration (artificial ovary): Promoted rapid engraftment to regenerate the uterine lining and mitigate menopause-related risks.	[83]
	Fibrin + VEGF	Beads	For fertility restoration (artificial ovary): Supported follicle transplantation, leading to resumed menstrual cycles and live births.	[90]
	Decellularized ovarian tissue (OECM)	Hydrogel	For post-chemotherapy infertility: Supported follicle engraftment in a bioengineered “in situ ovary”, resulting in healthy offspring.	[46]
		Scaffold	For fertility restoration (artificial ovary): Restored endocrine function and initiated puberty after recellularization and transplantation.	[91]
	Chitosan + Platelet lysate	Nanoparticle	For POI: Increased AMH levels, number of retrieved oocytes, and blastocyst formation rate.	[133]
	Growth hormone (GH)-loaded nanocomplex (ZIF8-GH@ZP3Ab)	Nanoparticle	For POI: Targeted follicles to deliver GH, restoring ovarian function and fertility by reducing oxidative stress.	[134]
	Iron chelator (DFO)-loaded nanoparticles (FSH-mPDA@DFO)	Nanoparticle	For POI: Targeted granulosa cells to regulate iron metabolism and inhibit ferroptosis, improving oocyte quality.	[135]
	Chitosan + Curcumin	Nanoparticle	For PCOS: Reduced serum levels of LH, testosterone, and insulin.	[147]
Uterus	Hyaluronic acid	Hydrogel	For IUA: Acted as a physical barrier to prevent post-surgical adhesion recurrence.	[157]
	Exosome	Hydrogel	For IUA: Promoted endometrial regeneration, leading to fertility restoration and live births in animal models.	[160]
	Porcine endometrial decellularized matrix (EndoECM)	Hydrogel	For IUA: Increased endometrial glands, promoted angiogenesis, and reduced fibrosis to restore fertility.	[23]
	Decellularized uterine tissue	Scaffold	For UFI: Successfully regenerated into functional uterine tissue after being re-seeded with cells and transplanted in animal models.	[194]
	Hyaluronic acid hydrogel + Apoptotic bodies	Hydrogel	For UFI (IUA): Used a hydrogel system to deliver MSC-derived apoptotic bodies, showing therapeutic potential for intrauterine adhesions.	[195]
	Aloe vera–alginate hydrogel + MSCs	Hydrogel	For UFI (injury): Alleviated maternal simulated birth injury through vaginal delivery of tissue-engineered endometrial stem cells in a hydrogel.	[197]
Others	PLGA + Chrysin	Nanoparticle	For endometriosis: Reduced lesion implantation by suppressing peritoneal inflammation and angiogenesis.	[182]
	Bacteria outer membrane + PLGA	Nanoparticle	For endometriosis: Modulated immune cells (macrophages) to suppress fibrosis and disease progression.	[187]

### Ovarian follicle culture in vitro

Ovarian follicle culture is a cornerstone of reproductive tissue bioengineering, providing a platform to preserve mature oocytes in vitro, especially for patients unable to undergo conventional ovarian stimulation, such as prepubertal girls or individuals with hormone-sensitive cancers [49]. Biomaterials play a pivotal role in reconstructing the ovarian microenvironment in vitro, preserving follicular architecture, and supporting the cell–cell interactions essential for successful oocyte maturation and steroidogenesis. The ovarian follicle exhibits a 3D architecture, with an oocyte encircled by granulosa cells (GCs) within a basement membrane and, at more mature stages, enveloped by theca cells (TCs). This spatial organization is critical for bidirectional communication that regulates oocyte development and hormonal functions. Conventional 2D culture systems disrupt this architecture, resulting in premature follicle flattening, impaired granulosa–oocyte communication, and early atresia [50]. To overcome these limitations, 3D culture systems employing hydrogels have emerged as a robust strategy to maintain follicular integrity and function.

**Fig. 2.**
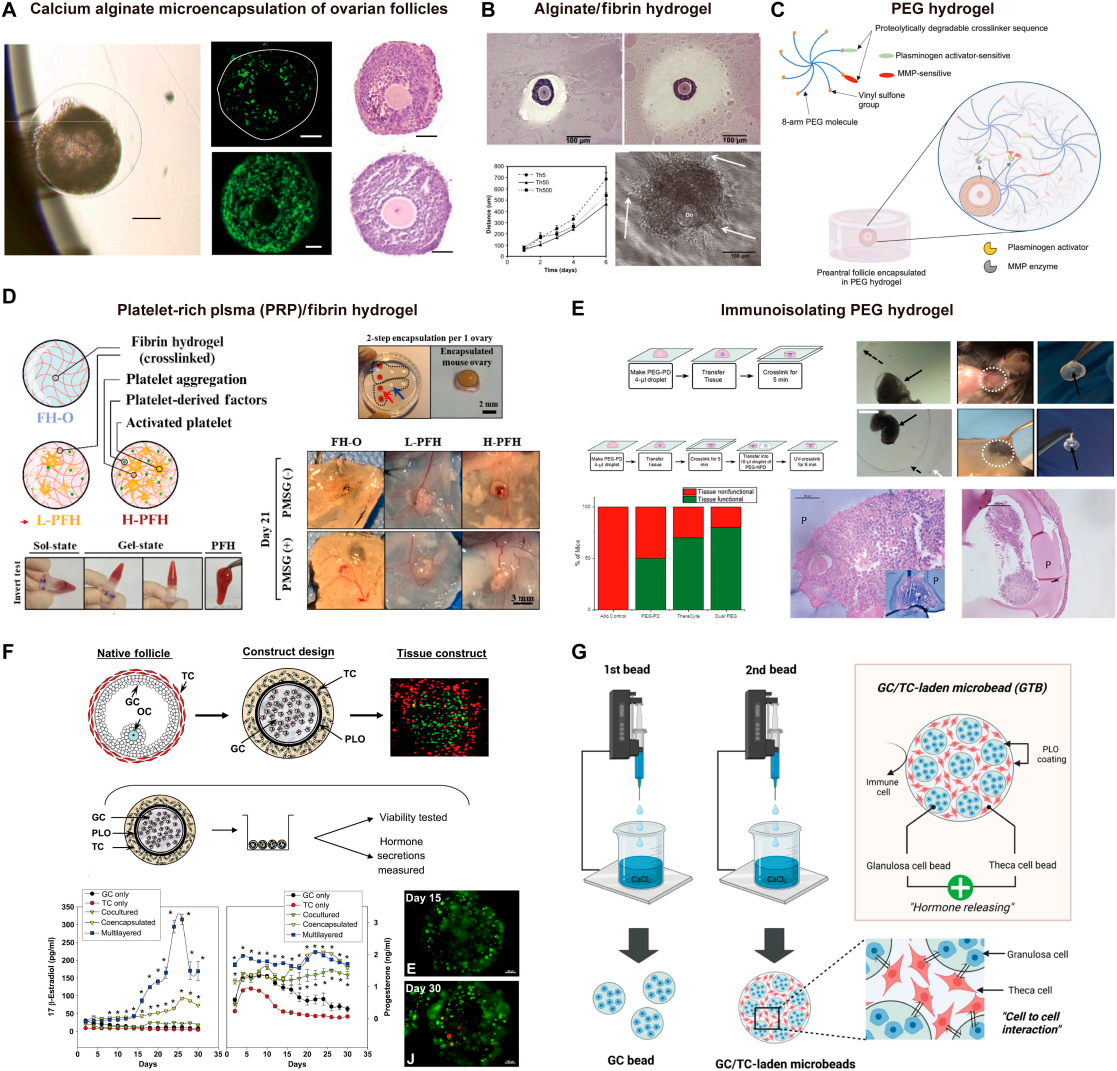
Biomaterials for in vitro ovarian follicle culture, ovarian tissue transplantation (OTT), and artificial ovaries for hormone production. (A) Calcium alginate microencapsulation of ovarian follicles. The images show a single murine follicle encapsulated within a calcium alginate bead, including brightfield, fluorescence viability staining, and histological analysis. (B) Composite alginate/fibrin hydrogel. Histological images and a growth curve show the successful development of an encapsulated ovarian follicle within an interpenetrating network of alginate and fibrin. (C) Enzymatically degradable PEG hydrogel. Schematic and images of a follicle encapsulated in a PEG hydrogel crosslinked with matrix metalloproteinase (MMP)-sensitive peptides, allowing for cell-mediated degradation and remodeling. (D) Platelet-rich-plasma (PRP)/fibrin hydrogel for whole ovary transplantation. A schematic of the PRP/fibrin hydrogel and in vivo results show follicular development on ovaries transplanted within different formulations of the hydrogel in a mouse model. (E) Application of a PEG hydrogel for immunoisolation. Demonstration of a PEG hydrogel used to encapsulate and immunoisolate transplanted cells, resulting in prolonged graft survival and function in vivo. (F) Design and hormonal function of a hierarchically structured artificial follicle. Left: A schematic of a native follicle, the design of a multilayered artificial follicle reconstructing granulosa cells (GCs) and theca cells (TCs) in a layered structure, and a viability stain image of the fabricated tissue construct. Right: Secretion of 17β-estradiol (E2) and progesterone over time from different cell groups (GC only, TC only, cocultured, coencapsulated, and multilayered). (G) Fabrication of ovary-mimic microbeads using microfluidics and subsequent vascularization. The fabricated GC/TC-laden microbeads (GTBs) secrete hormones via cell-to-cell interactions. Successful neovascularization at the implantation site is confirmed by the change in the E2/progesterone ratio and blood flow measurements over time. [Representative images reproduced adapted with permission from (A) [53] Springer Nature, (B) [55] Elsevier, (C) [64] Springer Nature, (D) [78] Elsevier, (E) [79] Springer Nature, (F) [85] Elsevier, and (G) [88] American Association for the Advancement of Science.]

Hydrogels derived from natural and synthetic polymers (e.g., alginate, fibrin, collagen, chitosan, PEG, and gelatin) have been widely explored for follicle encapsulation (Fig. 2A and B) [49,51–55]. Alginate is a naturally occurring polysaccharide that has been studied for its biocompatibility, permeability, and mild gelation conditions that preserve follicular architecture. However, its bioinert nature and lack of natural cell adhesion motifs impair GC–oocyte communication and limit long-term follicle maturation and ovulation [56]. Accordingly, alginate is often modified with adhesive peptides [e.g., arginylglycylaspartic acid (RGD)] or blended with ECM components to enhance cell–matrix interactions and better mimic the ovarian niche [57,58]. PEG-based hydrogels exhibit tunable mechanical properties and low immunogenicity, which aid in mimicking the dynamic mechanical and biochemical environments of the ovarian stroma. The inert backbone of PEG permits precise control over mechanical stiffness and degradation kinetics, thereby supporting regulated follicle expansion while minimizing immune activation [59,60]. Collagen hydrogels are commonly used for ovarian follicle culture due to their natural origin and bioactivity, which provide cell-adhesive ligands that support GC spreading and signaling. Nevertheless, native collagen gels exhibit limited mechanical tunability and may compact during long-term culture, affecting follicle growth. Joo et al. [54] reinforced collagen hydrogels with fibrin to enhance mechanical stability while preserving biocompatibility and supporting healthy follicular development and hormone production.

Moreover, matrix stiffness critically influences follicular development. The mechanical properties of biomaterial scaffolds are pivotal for regulating follicle growth during in vitro culture. A previous study determined that an intermediate stiffness (~0.5 to 1 kPa), reproducing the native ovarian cortex, supported optimal follicle development and hormone production [61]. Excessively soft matrices caused follicle collapse, whereas overly stiff scaffolds obstructed cell proliferation and nutrient transport. Choi et al. [62] demonstrated that a collagen–alginate bilayer scaffold engineered to replicate the stiffness gradient between the ovarian cortex and medulla substantially enhanced folliculogenesis. In the present work, the stiff outer alginate layer (cortex) provided structural confinement, and the soft inner collagen layer (medulla) permitted GC expansion and follicular maturation, thus enabling in vitro ovulation without exogenous luteinizing hormone (LH) and epidermal growth factor (EGF) stimulation. Kim et al. [63] identified that follicles cultured in an extracellular matrix-derived soft hydrogel (~60 Pa) exhibited superior pseudo-antrum formation and steroid hormone production compared to those in stiffer alginate (~800 Pa), underscoring the advantage of low-modulus matrices for folliculogenesis. Matrix degradability constitutes another key factor in long-term culture. Recently, Candelaria et al. [64] reported that incorporating matrix metalloproteinase (MMP)-sensitive linkers enabled scaffold remodeling in response to follicular expansion, supporting growth and antrum formation (Fig. 2C).

Although mechanical stiffness governed the structural aspects of follicle development, biochemical signaling remains essential for recapitulating the ovarian niche. Growth factors such as insulin-like growth factor (IGF), EGF, and kit ligands (KLs) promoted GC proliferation and oocyte maturation. Encapsulation matrices can be functionalized with ECM-derived peptides (e.g., RGD motifs) or tethered to localized growth factor reservoirs to more accurately mimic the ovarian niche. Follicle culture platforms are used primarily for fertility preservation in cancer patients at risk of ovarian failure owing to chemotherapy or radiotherapy. Araújo et al. [65] investigated in vitro development of ovarian follicles in 2D and 3D alginate-based culture systems supplemented with VEGF, IGF-1, and growth hormone (GH). Their results demonstrated that the 3D alginate system combined with these growth factors significantly enhanced follicle growth, antrum formation, and estradiol (E2) production, underscoring biomimetic environments’ advantages for folliculogenesis. Skory et al. [43] reported that murine follicles cultured in a 3D alginate hydrogel under gonadotropin stimulation underwent ovulation in vitro and replicated in vivo-like hormonal cycles encompassing follicular and luteal phases. The study further revealed that human follicles responded similarly to follicle-stimulating hormone (FSH), human chorionic gonadotropin (hCG), and EGF treatments, suggesting that engineered 3D culture systems can model the entire ovarian cycle. This approach established a physiologically relevant platform for studying folliculogenesis and hormonal dynamics ex vivo. Recent investigations examined interactions between stem cells and ovarian follicles to enhance in vitro folliculogenesis. Green et al. [17] demonstrated that coencapsulation of follicles with adipose-derived stem cells (ADSCs) in alginate hydrogels significantly improved follicle survival, growth, and antrum formation. Tomaszewski et al. [66] further demonstrated that 3D coculture with ADSCs in PEG-based matrices enhanced follicle development more effectively than conditioned media from 2D cultures, highlighting the significance of spatial paracrine signaling.

Advancing ovarian tissue engineering necessitates a rigorous evaluation of the inherent trade-offs among biomaterial classes. Natural polymers, such as collagen, supply physiologically relevant adhesion ligands and biochemical cues but are limited by batch-to-batch variability and poor mechanical tunability. In contrast, synthetic matrices like PEG provide highly precise and reproducible mechanical control, yet require targeted biochemical functionalization to overcome their intrinsic bioinert property. Although biomaterials such as alginate are continually optimized, deriving a quantitative consensus is difficult due to substantial variability in experimental parameters. Taken together, these observations revealed that the field is unlikely to converge on an optimal biomaterial. Instead, future progress will depend on the rational engineering of composite hydrogel systems that integrate the biochemical characteristic of natural polymers with the mechanical precision and reproducibility of synthetic scaffolds. Such hybrid constructs will enable finely controlled modulation of stiffness, viscoelastic properties, and degradation kinetics, thereby establishing the dynamically adaptive microenvironment required to recapitulate the complex, multistage process of folliculogenesis.

### Ovarian tissue transplantation

OTT involves the surgical grafting of fresh or cryopreserved ovarian tissue to restore fertility and endocrine function in women at high risk of premature ovarian insufficiency (POI) from gonadotoxic treatments such as chemotherapy and radiotherapy [67,68]. Compared to oocyte or embryo cryopreservation, OTT does not require ovarian stimulation, rendering it especially beneficial for prepubertal girls or patients needing urgent treatment. Despite clinical advances, ischemic damage and limited graft longevity persist as primary constraints in reestablishing ovarian function [69]. Typically, OTT entails reimplantation of thawed ovarian cortical fragments into orthotopic sites (e.g., the ovarian medulla or peritoneal pouch) or heterotopic locations (e.g., subcutaneous tissues in the forearm or abdominal wall). Recent developments in functional biomaterials have yielded innovative approaches to address these challenges by enhancing graft survival, promoting vascularization, and mitigating immunological and oncological risks. To counter ischemia-induced follicle loss during the early revascularization phase, biomaterials are designed to promote rapid angiogenesis and provide a bioactive, stromal-mimetic matrix that aids follicle viability.

Numerous studies have employed pro-angiogenic factors—including simvastatin, angiopoietin-2, VEGF, and basic fibroblast growth factor (bFGF)—to accelerate neovascularization, thus reducing ischemic injury following OTT [70–74]. Nonetheless, the rapid systemic clearance and short in vivo half-life of these molecules underscore the need for biomaterial-based delivery systems that sustain localized release. Shikanov et al. [75] engineered fibrin hydrogels modified with a heparin-binding peptide and loaded with VEGF for controlled delivery of angiogenic factors. This approach doubled primordial follicle survival and improved vascularization in a murine ovarian autotransplantation model, ultimately restoring fertility. Yang et al. [76] designed a fibrin hydrogel incorporating nitric oxide-releasing nanoparticles to promote angiogenesis around transplanted ovarian follicles; they demonstrated that this method significantly enhanced neovascularization and follicular survival. Similarly, natural polymer hydrogels containing bioactive factors, including hyaluronic acid (HA), collagen, gelatin, and alginate, are widely employed to restore ovarian function following transplantation [77]. For example, Chung et al. [78] reported a platelet-derived factor-encapsulated fibrin hydrogel (PFH) during OTT (Fig. 2D). By examining how the composition of platelet-derived factors in PFH influences vascular growth, their study sought to promote tissue repair and protect follicles. This research may enhance OTT and fertility preservation strategies.

Immune rejection remains a major concern in OTT, particularly when allogeneic or xenografts are used. Transplanted ovarian tissues can provoke host immune responses characterized by donor-specific antibody formation, T cell infiltration, and eventual graft failure. To address these risks, biomaterials such as PEG-based hydrogels have been explored as immunoisolation barriers. For example, encapsulating ovarian allografts within dual-layer PEG hydrogels in immunocompetent mice markedly reduced immune cell infiltration and maintained endocrine function beyond 60 d [60]. Day et al. [79] presented a dual-layer PEG-based hydrogel capsule for encapsulating ovarian allografts and evaluated its capacity to restore endocrine function in ovariectomized immune-competent mice (Fig. 2E). The study found that non-encapsulated grafts elicited strong alloimmune responses—evidenced by a 26-fold increase in donor-specific immunoglobulin G (IgG) and CD8^+^ T cell infiltration—whereas encapsulated grafts maintained endocrine function for 60 d without inducing immune sensitization or lymphocyte infiltration, thus demonstrating effective immunoisolation and hormonal restoration. Moreover, the findings indicated that the maturity of donor ovarian tissue influenced immune response severity, with mature tissue eliciting stronger alloimmunity than juvenile grafts [80]. Although immunoisolation strategies employing biomaterials have shown promising results in preclinical models, clinical translation requires overcoming significant hurdles to ensure long-term efficacy and safety. The primary challenge remains designing a multifunctional platform that provides robust immunoprotection while actively promoting rapid revascularization to prevent ischemic damage. Future research should optimize the physical properties of these barriers, such as pore size and permeability, to permit essential nutrient and hormone exchanges while effectively blocking immune cell infiltration. Furthermore, a deeper understanding of the dynamic host–graft immune dialogue is required to tailor immunomodulatory strategies, potentially by incorporating anti-inflammatory agents or engineering materials that modulate the local immune environment. Ultimately, long-term therapeutic success in allogeneic OTT will depend on integrating advanced immunoisolation techniques with proangiogenic strategies to establish a supportive and protective niche for transplanted ovarian tissue.

### Hormonal restoration with engineered artificial ovaries

Premature ovarian insufficiency (POI) and menopause precipitate both the loss of fertility and the abrupt cessation of ovarian hormone synthesis, yielding profound systemic effects. Although exogenous HRT remains the clinical standard, it poses several limitations. Oral formulations undergo extensive first-pass hepatic metabolism; alternative routes, such as transdermal patches or vaginal rings, exhibit inconsistent absorption and localized side effects [81,82]. Advanced nanoparticle-based platforms have been developed to enhance bioavailability; they cannot obviate the principal limitation shared by all exogenous therapies—a nonphysiological delivery pattern. Conventional HRT provides a continuous hormone supply yet fails to replicate natural pulsatile secretion or respond to endogenous feedback signals from the hypothalamus and pituitary gland. This nonbiomimetic approach may result in significant long-term risks, including an elevated incidence of thrombosis and hormone-sensitive malignancies.

Engineered “artificial ovaries” have emerged as transformative alternatives to address these challenges. These constructs encapsulate hormone-producing cells—such as GCs, TCs, or iPSC-derived ovarian cells—within a biocompatible scaffold. This biomaterial renders a 3D architecture that supports cell organization, facilitates nutrient diffusion, and shields cells from host immune responses. These engineered tissues respond to natural gonadotropin cues, enabling sustained, localized, and regulated hormone release, thus restoring the hypothalamic–pituitary feedback axis that exogenous HRT cannot replicate.

The development of artificial ovaries has progressed through several key stages. Initial proof-of-concept studies demonstrated that ovarian cells encapsulated within hydrogels (e.g., PEG) restored endocrine function in ovariectomized animal models. These constructs reestablished cyclic E2 secretion, resulting in antral follicle and corpora lutea formation and reduced serum FSH levels, thereby mimicking physiological feedback [45,83,84]. Moreover, some designs provided effective immunoisolation by entirely preventing donor-specific antibody formation and T cell infiltration in immunocompetent recipients [60].

To enhance the longevity and functionality of these systems, subsequent research developed more sophisticated biomimetic structures. For instance, multilayered alginate capsules were engineered to replicate native ovarian architecture. In addition, incorporating mesenchymal stem cells (MSCs) into these constructs enhanced hormone secretion, reversed uterine atrophy, and improved long-term efficacy (Fig. 2F) [85–87]. Recent innovations have prioritized clinical translatability and safety. A key advancement was the creation of injectable, microsized hydrogel beads permitting minimally invasive delivery. This system restored endocrine function in menopausal rats while avoiding mammary gland hyperplasia associated with conventional HRT (Fig. 2G) [88]. Building on this, the latest generation of artificial ovaries incorporates prevascularized cell spheroids that rapidly integrate with host tissue, supporting robust, localized hormone release. Studies demonstrated full regeneration of the uterine lining and mitigation of menopause-related risks—osteoporotic weight gain, deep vein thrombosis, and metabolic derangements—without inducing systemic side effects of pharmaceutical hormones, representing a significant step toward a viable implantable alternative to HRT [83].

In addition, the implanted vascularized hydrogel with ovarian spheroids (VHOS) constructs restored estrogen-dependent tissue structure and function (e.g., endometrial regeneration and prevention of osteoporotic weight gain) while mitigating typical menopause risks, tissue overgrowth, hyperplasia, deep vein thrombosis, and metabolic derangements, thus indicating a viable alternative to conventional hormone replacement. Although bioengineered artificial ovaries restored physiological hormone cycles in preclinical models, the subsequent challenge remained attaining complete and lifelong endocrine function. Future research should emphasize the long-term viability and stability of encapsulated cells, necessitating development of dynamic biomaterials capable of active remodeling and adaptation. Scalable production of reliable hormone-producing cells such as induced pluripotent stem cells (iPSCs) under stringent safety protocols to prevent tumorigenicity remains a critical challenge. Ultimately, the objective is to develop a fully integrated artificial ovary that provides safe, feedback-responsive hormone therapy and mimics the natural progression of ovarian function, thereby bridging the gap between replacement and restoration.

### Artificial ovaries for fertility and live birth

**Fig. 3.**
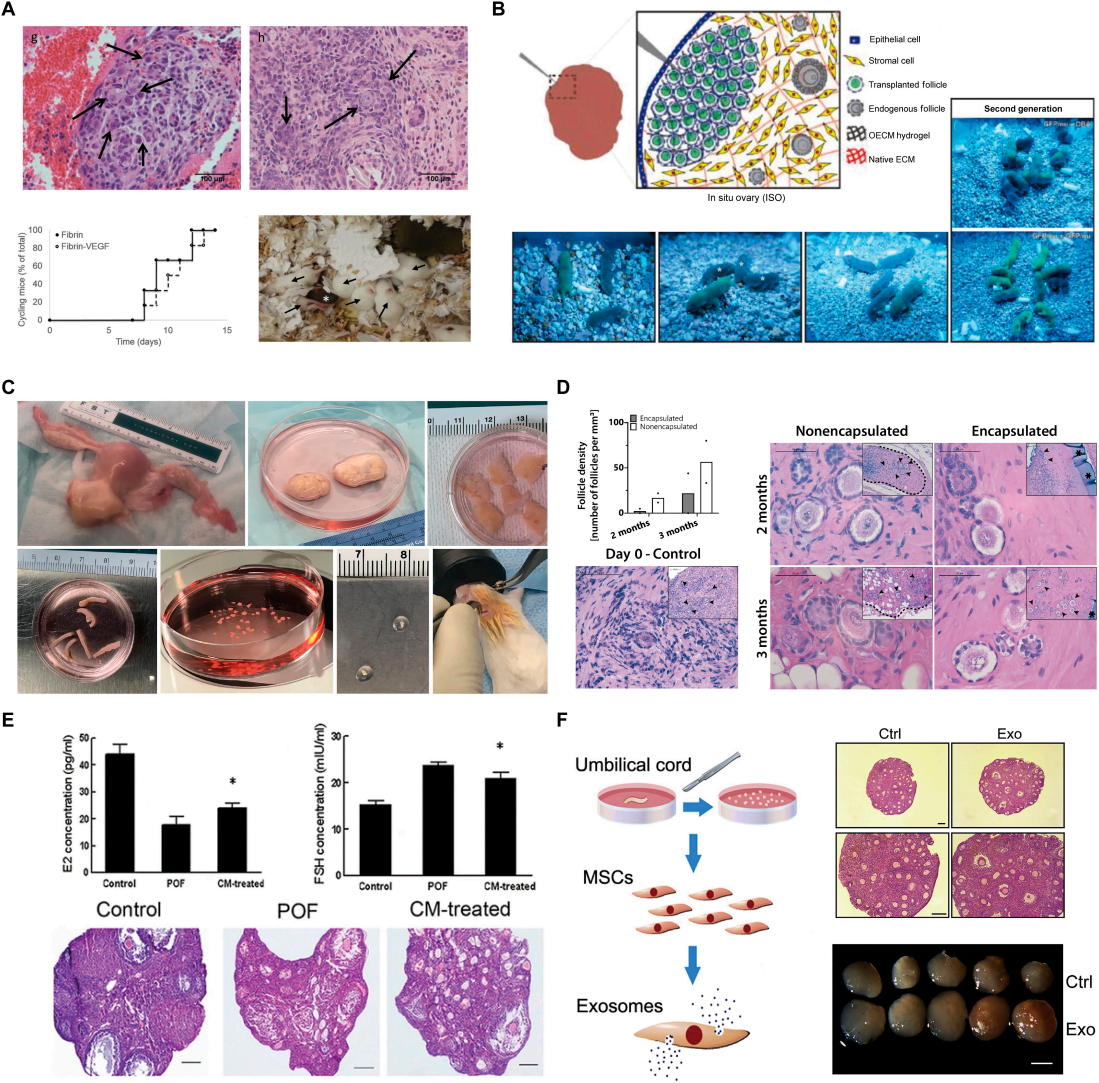
Preservation and restoration of ovarian function using hydrogel-based artificial ovary technology and cell-derived materials. (A) OTT using a fibrin hydrogel containing vascular endothelial growth factor (VEGF). Histological images show neovascularization (top, arrows) within the transplanted tissue. (B) “In situ ovary” concept and follicle development. A schematic diagram illustrates the concept of transplanting isolated follicles within a hydrogel onto the ovarian surface, along with images showing the growth and development of these transplanted follicles over time. (C) Follicle isolation, encapsulation, and transplantation procedure. A series of photographs showing the entire step-by-step process, from ovarian tissue collection, follicle isolation, encapsulation into hydrogel beads, to transplantation into an animal model. (D) Comparison of post-transplantation follicle survival with hydrogel encapsulation. The encapsulated follicles maintain a higher survival density (graph) and a more stable histological structure at 2 and 3 months post-transplantation compared to non-encapsulated follicles. (E) Therapeutic effect of conditioned medium (CM) on premature ovarian failure (POF). (Top) CM treatment restores E2 and follicle-stimulating hormone (FSH) levels in a POF model. (Bottom) Histological images show recovery of follicular development in the ovaries of CM-treated animals compared to the POF group. (F) Ovarian function recovery by transplantation of umbilical cord MSC-derived exosomes (Exo). A schematic shows the isolation of exosomes from mesenchymal stem cells (MSCs). Histological and macroscopic images show improved follicular structure and ovarian size in the exosome-treated group compared to the control (Ctrl). [Representative images reproduced adapted with permission from (A) [90] Springer Nature, (B) [46] SAGE, (C and D) [107] Frontiers, (E) [117] Springer Nature, and (F) [125] Cell Press.]

These efforts aim to overcome limitations inherent in current fertility preservation strategies for patients with therapy-induced ovarian failure, particularly in the pediatric oncology population, where cryopreserved OTT poses a significant risk of reintroducing malignant cells [84]. Conventional HRT fails to fully restore the complex endocrine and reproductive functions of the ovary, thereby limiting its capacity for offspring production. Consequently, there is an imperative to engineer artificial ovaries that support both follicular development and hormone production while mitigating the risk of cancer cell reintroduction. A promising strategy entails culturing ovarian follicles within biomaterial matrices. In particular, alginate-based hydrogels have been extensively employed to preserve the 3D follicular architecture and sustain critical cell–cell interactions in vitro [89]. This 3D culture system faithfully recapitulated the native follicular microenvironment, supporting the development of mature oocytes that demonstrated fertilization capacities comparable to in vivo matured oocytes. Embryos derived from in vitro fertilized oocytes were transferred into pseudopregnant female mice, resulting in viable, fertile male and female offspring. Kniazeva et al. [90] investigated ovarian follicle transplantation using biomaterial grafts for fertility preservation (Fig. 3A). They isolated primordial and primary ovarian follicles from young female mice, encapsulated them in various biomaterials—including fibrin, fibrin/alginate, and fibrin/collagen—and transplanted them into adult mice. Live births occurred exclusively in mice receiving VEGF-loaded fibrin beads, whereas all mice with fibrin-encapsulated follicles resumed menstrual cycles. The procedure also significantly reduced the metastatic breast cancer cell count, indicating a diminished risk of reseeding the disease compared to whole ovary transplantation. Despite producing viable offspring, its efficiency remained low. These findings underscore the necessity of incorporating functional biomaterials to support follicular development, vascularization, and sustained reproductive function.

One study examined the creation of a decellularized ovarian scaffold that was subsequently recellularized with healthy ovarian cells and transplanted into mice to induce puberty [91]. In this study, human and bovine ovaries underwent sodium dodecyl sulfate (SDS)-mediated decellularization, which removed cellular material, including SALL4-positive cancer cells, while preserving the native ECM microstructure. Primary mouse ovarian cells were seeded onto these scaffolds, and in vitro viability, follicle-like structure formation, and hormone production were assessed. Following in vivo transplantation into prepubertal ovariectomized mice, the recellularized grafts restored endocrine function. Serum E2 and inhibin A levels returned to near-normal concentrations, and puberty was initiated, evidenced by vaginal opening within 4 weeks. The decellularized scaffold also supported the growth of small follicles into large antral follicles while eliciting a minimal immune response. This methodology presents a promising and safer alternative to conventional OTT, particularly for patients at risk of cancer reintroduction, marking a significant advancement in artificial ovary development. Buckenmeyer et al. [46] developed a bioengineered “in situ ovary” (ISO) using an ovary-specific ECM (OECM) hydrogel to facilitate follicle transplantation (Fig. 3B). The approach aimed to address infertility in women undergoing chemotherapy. Using a hydrogel-based microinjection approach, researchers demonstrated that follicles transplanted into the ovaries of chemotherapy-treated mice were successfully engrafted and survived. Mice produced healthy offspring, demonstrating the effectiveness of the method. This technology offers a minimally invasive approach to restore fertility in female cancer patients.

Beyond isolated follicles, the cellular constituents of artificial ovaries are critical. GCs and TCs are essential hormone-producing cells; their spatial arrangement within the construct is vital for optimal function. Stem cell technology shows promise in generating new oocytes and supporting ovarian function. Pluripotent stem cells (PSCs), including iPSCs, have been differentiated into primordial germ cell-like cells (PGCLCs) and granulosa-like cells. When coencapsulated in appropriate scaffolds, these cell types partially restored endocrine and reproductive functions in vitro and in vivo. Notably, this system does not require embryonic ovarian tissue; thus, it could be applied to other mammalian species, providing an alternative source of oocytes for reproductive research and breeding [92]. This study challenges the common belief that female mammals lose their ability to produce eggs at birth. Researchers successfully isolated female germline stem cells (FGSCs) from newborn mouse ovaries and cultured them for over 15 months. After FGSCs were transduced with green fluorescent protein (GFP) virus and transplanted into the ovaries of sterile mice, the cells underwent oogenesis, and the mice gave birth to offspring expressing the GFP transgene. This study demonstrated that FGSCs could be used to produce offspring from sterile recipients. These findings open new avenues for application of stem cells in regenerative and anti-aging medicine.

### Immunomodulatory biomaterials in ovarian transplantation

Immune rejection remains a critical barrier to long-term success of OTT irrespective of biomaterial use. Cryopreservation of ovarian tissue collected prior to gonadotoxic therapy preserves fertility and endocrine function following transplantation. Nevertheless, the presence of malignant cells in preserved ovarian tissue poses a significant oncological risk, thereby limiting clinical applicability [84,90,93]. Encapsulating donor ovarian tissues or follicles within immunoisolated capsules presents a promising alternative. Hydrogel-based immunoisolation offers high biocompatibility and water content, generating mechanical properties akin to the native ECM. Precisely controlling crosslinking density, polymer molecular weight, and concentration governs swelling, mesh size, and diffusivity, thus optimizing graft viability and function [94–97]. Widely used hydrogels for cell and tissue encapsulation include natural polymers such as alginate, chitosan, agarose, and fibrin, as well as synthetic types like PEG [98–101]. Hydrogels are valued for replicating the native ECM and providing biological and mechanical cues that influence cellular behavior.

Furthermore, transplanting hormone-producing cells within immunoisolated capsules has been investigated in type I diabetes, employing pancreatic islets as a therapeutic model [102–105]. Hormone-secreting cells, including ovarian follicles that function similarly to pancreatic islets by producing hormones in response to systemic signals (e.g., FSH), can be encapsulated within semipermeable membranes that permit nutrient and waste exchange while preventing immune cell contact, thereby reducing host rejection. Unlike islets, however, follicles are dynamic structures in which only a subset activates each cycle and undergoes considerable volumetric expansion. Consequently, an immunoisolating capsule for follicle encapsulation must provide immunoprotection while accommodating this growth. This requirement necessitates the use of a degradable or mechanically adaptive matrix that enables matrix remodeling during folliculogenesis. Follicles are naturally avascular and resilient to hypoxia, rendering them amenable to encapsulation strategies that rely solely on passive diffusion through the matrix for nutrients and oxygen.

David et al. [106] evaluated the restoration of ovarian endocrine function in ovariectomized mice by transplanting allogeneic ovarian tissue encapsulated in alginate capsules or TheraCyte, with the latter enabling follicular growth while effectively isolating the graft from host immune recognition. Recent studies have thus focused on immunomodulatory biomaterials capable of actively directing local immune responses rather than relying solely on passive immunoisolation. Brunette et al. [107] addressed POI by encapsulating human ovarian tissue in PEG-based hydrogels, which maintained follicle survival after implantation in mice at levels comparable to non-encapsulated grafts. This approach exhibited long-term viability, with follicles in implanted vitrified tissues surviving for 3 months in vivo. Furthermore, the transplanted tissue maintained endocrine function: E2 levels remained detectable for 21 d post-implantation, thus suggesting restoration of hormone production in patients (Fig. 3C and D). Although physical immunoisolation with hydrogels such as dual-layer PEG capsules blocked host immune cell infiltration and maintained endocrine function in murine models [60], this strategy may prove insufficient to address the complexity of human immune responses.

Earlier sections examined the mechanical and bioactive functions in folliculogenesis. Notably, dECMs display low immunogenicity in transplant models owing to absent donor cellular antigens and retained tolerogenic ECM components [34]. Liu et al. [108] demonstrated that decellularized ovarian tissues were noncytotoxic to rat ovarian cells in vitro and elicited only a minimal immunogenic response in vivo. Moreover, decellularized ovarian tissues supported rat GC infiltration ex vivo and enhanced E2 secretion. Recently, Liu et al. [109] proposed treating POI with hydrogel microspheres (OG-HMPs) comprising decellularized OECM and gelatin to deliver bone marrow-derived mesenchymal stem cells (BMSCs). OG-HMPs provided a scaffold that enhanced BMSC adhesion and proliferation, facilitating dense spheroid formation. Preserved ovary-specific bioactive cues in OECM reconstituted a tissue-relevant microenvironment, improving cell viability and markedly up-regulating proangiogenic factors VEGF and hepatocyte growth factor (HGF). Furthermore, OG-HMPs loaded with BMSCs exerted immunoregulatory effects on activated macrophages and protected GCs from chemically induced apoptosis. ECM-derived scaffolds, notably decellularized ovarian or reproductive matrices, exhibit intrinsic immunomodulatory properties by retaining bioactive molecules and structural elements that promote immune quiescence [48,110].

Recent advances in hydrogel-based encapsulation, dECM scaffolds, and stem cell delivery platforms have shifted emphasis from passive immunoisolation to active immunomodulating biomaterial systems. Researchers have integrated controlled-release approaches, immunoregulatory signals, and stimulus-responsive scaffold architectures to modulate the host immune milieu, thereby supporting graft survival and integration. Incorporating patient-specific immune profiles into biomaterial design could enhance therapeutic efficacy, enabling next-generation immunomodulatory platforms that accommodate diverse immune landscapes in ovarian transplantation and artificial ovary applications.

## Engineering Functional Biomaterials for Overcoming Reproductive Dysfunctions

Reproductive disorders—including premature ovarian insufficiency (POI), IUAs, PCOS, endometriosis, and UFI—remain major causes of female infertility worldwide. These conditions compromise the structural and functional integrity of reproductive tissues, resulting in hormonal imbalances, impaired gametogenesis, and disrupted embryo implantation. Although pharmacological and surgical interventions remain first-line therapies, functional biomaterials offer emerging strategies for repairing, regenerating, or restoring reproductive function by modulating the local tissue microenvironment, enabling cell recruitment, hormone delivery, immune regulation, and tissue reconstruction.

### Primary ovarian insufficiency

The strategies discussed—spanning from in vitro ovarian follicle culture to artificial ovary bioengineering—address POI by supporting follicular survival and stimulating residual ovarian function; POI describes premature loss of ovarian function before age 40, leading to infertility, hypoestrogenism, and elevated gonadotropin levels [111]. Genetic, autoimmune, and iatrogenic factors, such as chemotherapy and radiotherapy, may contribute to POI. Current POI therapies, including conventional HRT and ovarian tissue cryopreservation, exhibit limitations, such as an elevated ovarian cancer risk and potential malignant-cell reintroduction. Here, we review cell- and biomaterial-based strategies for POI that aim to restore function rather than regenerate organ tissues.

Stem cell-based regenerative strategies have emerged as promising for repairing damaged or dysfunctional tissues. Among these, MSCs are widely used in regenerative medicine and offer therapeutic potential for POI [112–115]. Numerous molecular studies have confirmed the capacity of MSCs to restore ovarian function by elucidating mechanisms via signaling pathways, gene regulation, and paracrine factor secretion. In both in vitro and in vivo POI models, MSCs modified protein expression profiles, modulated the estrous cycle, normalized sex hormone levels, and enhanced fertility. Moreover, MSC-based cell-free therapies, such as secretomes, cytokines, and exosomes, are investigated as safer alternatives that circumvent immunogenicity and tumorigenicity [116]. In a POI rodent model, menstrual-derived stem cells (MenSC)-conditioned medium administration reduced serum FSH, increased E2, enhanced ovarian function, reduced fibrosis, and decreased apoptosis in ovarian GCs (Fig. 3E) [117]. Similarly, Khanmohammadi et al. [118] reported no significant differences in ovarian structure or serum hormone profiles were observed between POI rats treated with bone marrow-derived mesenchymal stem cells (BMMSCs) and those receiving a BMMSC-derived secretome. Multiple studies have examined stem cell-derived exosomes as potential POI therapeutics and elucidated their mechanisms of action [119–122]. Human umbilical cord MSCs secrete cytokines that enhance ovarian expression of HGF, VEGF, and IGF-1, thereby restoring function and delaying senescence, and human umbilical cord mesenchymal stem cells (HucMSC)-derived exosomes modulate gene and protein expression to regulate apoptosis in ovarian granulosa cells (OGCs) [123,124]. Furthermore, Yang et al. [125] confirmed that these exosomes activate primordial follicles and promote oocyte development; administration to aged mice (>10 months) restored fertility and improved both oocyte quantity and quality (Fig. 3F). Xiao et al. [126] determined that miR-10a from amniotic fluid stem cell (AFSC)-derived exosomes suppressed apoptosis in OGCs from patients with POI by down-regulating specific genes in the SMAD signaling pathway.

To enhance therapeutic efficacy, researchers incorporated these regenerative agents into biocompatible materials and engineered ovarian patches that provide structural support and enable sustained delivery, thereby promoting follicular development and functional recovery [127]. Biomaterial design depends on factors such as fabrication method, biocompatibility, and capacity to support favorable cellular interactions [128–130]. An ideal biomaterial should be nontoxic, biocompatible, biodegradable, and bioresorbable, permitting tissue regeneration without eliciting inflammatory responses [131]. The effectiveness of these materials in reversing ovarian dysfunction is further discussed in subsequent sections [132]. A study investigated the therapeutic efficacy of platelet lysate-loaded chitosan nanoparticles (PLCH NPs) in a cyclophosphamide (CP)-induced POI mouse model [133]. Prepared via ionic gelation, PLCH NPs exhibited sustained release, with >97% platelet lysate (PL) released within 48 h. POI mice treated with PLCH NPs exhibited elevated anti-Müllerian hormone (AMH) and total anti-oxidant capacity (TAC) levels alongside reduced malondialdehyde (MDA) levels. Compared with the premature ovarian failure (POF) group, the POF-PLCH group yielded more oocytes and higher cleavage, fertilization, and blastocyst formation rates. Yang et al. [134] developed a follicle-targeting nanocarrier for GH delivery to restore ovarian function. The ZIF8-GH@ZP3Ab nanocomplex, comprising GH-loaded zeolitic imidazolate frameworks-8 (ZIF8), restored ovarian function and fertility in a cisplatin-induced POI mouse model by promoting follicle development and attenuating oxidative stress and apoptosis.

**Fig. 4.**
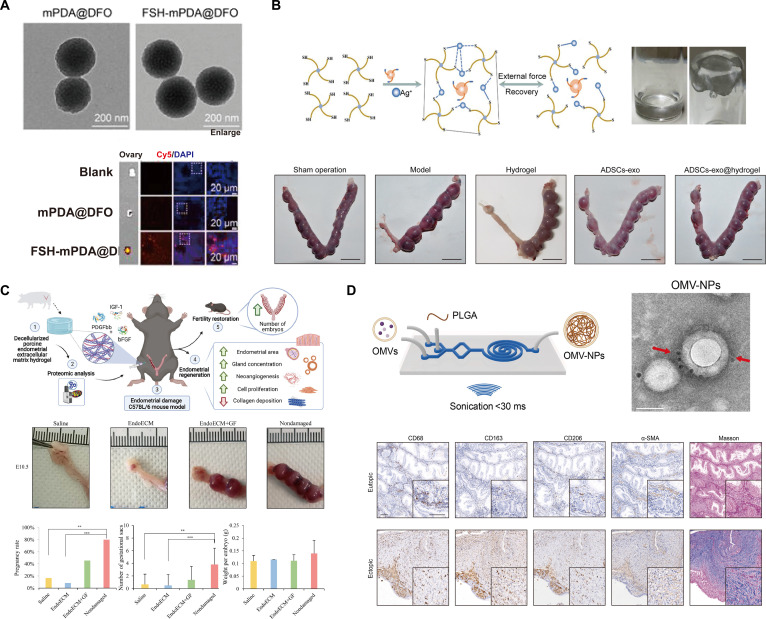
Nanodelivery systems for treating ovarian dysfunction and uterine repair and regeneration. (A) Ovary-targeting drug delivery system using FSH-functionalized nanoparticles. Transmission electron microscopy (TEM) images show the morphology of nanoparticles before (mPDA@DFO) and after (FSH-mPDA@DFO) FSH functionalization. In vivo fluorescence imaging demonstrates the specific accumulation of FSH-functionalized nanoparticles in the ovary. (B) Self-healing hydrogel loaded with adipose-derived stem cell exosomes (ADSCs-exo) for treating IUAs. The schematic (top) illustrates the injectable, self-healing properties of the hydrogel. Macroscopic images of uteri (bottom) from different treatment groups show that the ADSCs-exo@Hydrogel formulation most effectively promotes uterine regeneration in a damaged model. (C) Decellularized endometrial matrix hydrogel (EndoECM) for endometrial regeneration and fertility restoration. The illustration (top) shows the preparation of EndoECM and its application in a mouse model of endometrial damage. The hydrogel treatment restored uterine structure and function, leading to significantly improved pregnancy rates and number of viable pups compared to other groups. (D) PLGA-coated outer membrane vesicle nanoparticles (OMV-NPs) for immunomodulation and anti-fibrosis therapy in the endometrium. The top schematic shows the microfluidic fabrication and core–shell structure of OMV-NPs. Immunohistochemical staining (bottom) for macrophage markers (CD68, CD163, CD206) and fibrosis markers (α-SMA, Masson’s trichrome) suggests the nanoparticles promote an anti-inflammatory and anti-fibrotic environment. [Representative images reproduced adapted with permission from (A) [135] Ivyspring, (B) [160] Wiley, (C) [23] Elsevier, and (D) [187] Elsevier.]

Recently, Zhang et al. [135] prepared FSH-mPDA@DFO nanoparticles by encapsulating the iron chelator deferoxamine (DFO) within mesoporous polydopamine (mPDA) and conjugating an FSHβ peptide for specific targeting of ovarian GCs (Fig. 4A). These nanoparticles targeted GCs, reduced intracellular iron uptake, modulated iron metabolism, prevented mitochondrial damage, and decreased reactive oxygen species (ROS) production to avert ferroptosis. In vivo, POI-induced mice treated with these nanoparticles exhibited increased oocyte number and quality, and restored reproductive capacity and endocrine homeostasis.

In summary, integrating stem cell- and biomaterial-based strategies offers a promising alternative to address limitations of conventional POI treatments. The capacity of stem cells and their derivatives to restore ovarian function, along with structural support and sustained delivery from biocompatible materials and targeted nanoparticles, exhibits considerable potential. Future research on these complex, multifaceted approaches will be crucial for restoring ovarian function and improving quality of life in POI patients.

### Polycystic ovary syndrome

PCOS is the leading cause of endocrine and reproductive dysfunction in women. In contrast to POI, which is characterized by follicle loss, PCOS constitutes a multifaceted metabolic disorder driven by a cycle of insulin resistance and hyperandrogenism [136]. Hormonal disruption impairs folliculogenesis, resulting in ovulatory dysfunction and the accumulation of immature follicles. The 2 primary pharmacological treatments for PCOS target distinct aspects of the disease (hyperandrogenism and insulin resistance) and may adversely affect one another, generating clinical challenges [137]. Clinicians treating PCOS patients must choose whether to prioritize hyperandrogenism management with combined oral contraceptive pills (COCPs) or focus on metabolic health with metformin. Whereas COCPs may alleviate cosmetic symptoms, they risk exacerbating the long-term metabolic profile (insulin resistance and cardiovascular risk) [138]. Metformin can address the underlying metabolic issues, yet may not adequately control hyperandrogenic symptoms and is often poorly tolerated [139]. Although combination therapy is possible, it merges the adverse profiles of both drugs [140]. This therapeutic paradox highlights an unmet need for treatments that simultaneously and locally address both hormonal and metabolic dysregulation within the ovary without systemic side effects. These findings provide a rationale for nanoparticle (NP)-based systems for targeted ovarian drug delivery. Direct administration to the ovary can interrupt the cycle of insulin resistance and hyperandrogenism, thereby enhancing efficacy and diminishing systemic risks, such as venous thromboembolism (VTE) or gastrointestinal distress. Accordingly, recent studies evaluated biomaterials as localized delivery platforms for insulin sensitizers, anti-androgenic agents, and gonadotropins to modulate ovarian function [141–143].

Nanobiomaterials provide an innovative approach for PCOS treatment by enabling site-specific, controlled drug delivery that addresses pathology directly within the ovary [144–146]. By targeting granulosa and TCs, nanoparticles regulate steroidogenesis, improve insulin sensitivity, enhance drug bioavailability, and protect therapeutic agents from degradation, thereby reducing dosages and mitigating systemic toxicity [147]. This strategy has been implemented on several platforms. For example, natural polymer systems—such as chitosan nanoparticles loaded with curcumin—have effectively overcome curcumin’s low solubility by reducing serum LH, testosterone, and insulin levels in rat models [147]. Metal and metal oxide nanoparticles have also demonstrated potential; selenium nanoparticles reduce androgen synthesis and improve insulin sensitivity by modulating phosphatidylinositol 3-kinase (PI3K)/Akt signaling, whereas zinc oxide nanoparticles increase E2 production and elicit an antioxidant response. Moreover, lipid-based nanoparticles such as liposomes deliver resveratrol derivatives and enhance their bioavailability and regulatory effects on steroid hormone secretion. Advanced carbon-based nanocarriers such as PEGylated graphene oxide quantum dots (GOQD-PEG) facilitate the sustained release of conventional drugs such as metformin and restore glucose uptake in in vitro insulin resistance models. These systems deliver a broad range of therapeutic payloads, encompassing conventional agents (e.g., metformin and clomiphene citrate) and natural compounds (e.g., curcumin, ginger, cinnamon, and resveratrol) [148,149].

PCOS pathogenesis is closely linked to chronic low-grade inflammation, which induces both insulin resistance and hyperandrogenism; consequently, compounds with potent anti-inflammatory properties—such as curcumin—constitute promising therapeutic candidates. Curcumin’s efficacy in reducing insulin resistance and its anti-androgenic effects are well documented, rendering it a strong option for addressing the multifaceted nature of PCOS. Nevertheless, its clinical application remains limited by inherent hydrophobicity, which results in poor stability and low bioavailability in aqueous environments. Nanotechnology-based delivery systems have therefore been developed to overcome these constraints. For example, Raja et al. [150] engineered a novel nanocarrier by modifying chitosan with arginine (Arg) and N-acetyl histidine (NAcHis) to form the conjugate Arg-CS-NAcHis. This conjugate self-assembled into nanoparticles in aqueous solution, yielding a stable vehicle for efficient encapsulation of hydrophobic curcumin. This strategy enhanced curcumin’s stability and delivery potential, thus offering a viable approach for harnessing its anti-inflammatory and anti-androgenic benefits in PCOS therapy.

### IUA and Asherman’s syndrome

Asherman syndrome represents a severe form of IUA, an acquired condition characterized by scar tissue formation (adhesions or fibrosis) within the uterine cavity. The primary etiology is damage to the endometrial basal layer, most frequently caused by dilatation and curettage (D&C) after childbirth or miscarriage [151]. Other etiologies include hysteroscopic surgery, genital tuberculosis, and chronic inflammatory responses. The fibrous adhesions reduce uterine cavity volume, yielding hypomenorrhea (light periods), amenorrhea (absent periods), cyclic pelvic pain, recurrent miscarriage, and infertility, all of which markedly impair quality of life [152].

Standard treatment involves hysteroscopic adhesiolysis, comprising surgical resection of scar tissue to restore anatomical structure [153]. Nevertheless, this approach can inflict further endometrial trauma, thereby triggering a cycle of elevated re-adhesion. Reports indicate recurrence rates of 30% in mild-to-moderate cases and over 62.5% in severe cases [154]. Even after successful surgery, pregnancy success rates remain low (22.5% to 40%). This scenario underscores the clinical paradox that therapeutic intervention can perpetuate pathology, as Asherman syndrome extends beyond the physical barrier imposed by adhesions. Damage to the basal layer housing the stem and progenitor cells responsible for cyclic endometrial regeneration represents the underlying issue [155]. Although hysteroscopic surgery removes scar tissue, it does not restore the regenerative capacity of the damaged basal layer. Consequently, the uterine interior remains unsuitable for implantation, explaining why merely preventing re-adhesion fails to improve pregnancy rates. Accordingly, the therapeutic aim must shift from adhesion prevention to regenerating a functional endometrial microenvironment. This paradigm supports the development of functional, biomaterial-based strategies with regenerative capabilities.

Early biomaterial approaches employed a biodegradable intrauterine barrier to physically separate the uterine walls during the critical 3- to 7-d post-surgery healing period to prevent recurrence [156]. These strategies utilized materials such as crosslinked HA-based gels (e.g., Hyalobarrier), poloxamer-based thermosensitive gels, and solid films such as Seprafilm (HA/carboxymethylcellulose) or Interceed (oxidized regenerated cellulose) [157]. Unlike traditional intrauterine devices (IUDs) or Foley catheters, these biodegradable materials eliminate the need for secondary removal and reduce infection risk [158]. Injectable hydrogels—liquid at room temperature and gelled at body temperature—are advantageous for filling uterine cavities of various shapes and ensuring complete coverage of the damaged surface [159]. For example, Lin et al. [160] designed a versatile exosome–hydrogel system that creates a protective microenvironment to support endometrial repair and fertility restoration in offspring following in situ microinjection (Fig. 4B). Similarly, advanced systems have been engineered: immunomodulatory platelet-rich plasma (PRP) double-network hydrogels [161]; injectable thermosensitive chitosan–lignin/poloxamer hydrogels for sustained PRP release [162]; and multi-responsive GelMA/alginate–Fe_3_O_4_ microcapsules permitting magnetic guidance and ultrasound-triggered therapy [163]. However, passive barriers present fundamental limitations. Multiple studies and meta-analyses demonstrated that although these materials effectively reduce re-adhesion rates, they do not decisively improve pregnancy or live birth outcomes [164]. This shortcoming arises from their failure to address the “barren ground” limitation, prompting the development of more functional, bioactive systems.

To achieve true regeneration, next-generation strategies employ scaffolds as templates to guide organized regrowth of functional endometrial tissue [165]. An ideal scaffold combines biocompatibility and biodegradability with an interconnected porous structure supporting cell infiltration, nutrient diffusion, and angiogenesis [166]. Natural polymer-based scaffolds are particularly valuable for replicating the endometrial microenvironment. Collagen is a major component of the uterine ECM, and its intrinsic properties facilitate cell adhesion and proliferation [167]. Clinically, collagen scaffolds have delivered bone marrow-derived stem cells into the uterus, inducing endometrial growth and yielding a successful pregnancy [168]. Notably, dECM derived from porcine endometrial tissue, which preserves tissue-specific structural proteins and latent growth factors, demonstrates potent regenerative effects. An injectable hydrogel form of porcine-derived endometrial dECM (EndoECM) increased endometrial gland density, promoted angiogenesis, reduced fibrosis, and ultimately restored fertility in rat models (Fig. 4C) [23]. In contrast, synthetic polymer scaffolds, such as polylactic-co-glycolic acid (PLGA) and polyglycerol sebacate (PGS), permit precise control over mechanical properties and degradation rates. Conversely, they lack the bioactivity of natural polymers and may require additional functionalization to promote cell adhesion.

The most advanced approach combines the physical barrier function of hydrogels with their reservoir capacity for localized, sustained release of therapeutic agents. This paradigm shift converts hydrogels from passive barriers into active regenerative hubs. MSCs derived from bone marrow (BMSCs), umbilical cord (UC-MSCs), and menstrual blood (MenSCs) are the most extensively investigated cell sources [169]. They exert effects primarily via paracrine signaling, secreting growth factors, cytokines, and exosomes that promote angiogenesis, inhibit fibrosis, modulate immune responses, and recruit endogenous progenitor cells. Delivery of MSCs within a hydrogel scaffold, such as collagen, HA, or Pluronic F-127, significantly enhanced their survival and retention in the uterine cavity compared to direct injection. This approach improved outcomes in preclinical models and clinical cases, including increased endometrial thickness and restored fertility. To address safety and regulatory challenges of live-cell therapy, including immunogenicity and tumorigenicity risks, researchers explored delivery of MSC-derived therapeutic factors—secretome, exosomes, and apoptotic bodies—via biomaterials. HA hydrogels have effectively delivered the MSC secretome and restored the morphology and function of damaged endometrium in a rat model. Moreover, hydrogels can be loaded with specific biomolecules to precisely modulate the tissue repair processes [170]. Angiogenic factors such as VEGF and bFGF restore the vascular supply essential for a healthy endometrium [171]. Hormones such as 17β-E2 may be incorporated to promote endometrial proliferation and modulate the local immune milieu. Additionally, strategies recruiting endogenous stem cells to the injury site by delivering homing factors such as stromal cell-derived factor-1α (SDF-1α) via a chitosan–heparin hydrogel are under investigation.

These advances suggest that biomaterials function as more than passive carriers of therapeutic agents. The most sophisticated biomaterial systems serve as “orchestra conductors”, holistically directing the complex processes of endometrial healing. For instance, an MSC-loaded dECM hydrogel system acts sequentially across multiple stages [172]. Immediately after surgery, it forms a physical barrier preventing re-adhesion. During the initial healing phase, anti-inflammatory and proangiogenic factors released from the MSCs and dECM inhibit fibrosis and initiate vascular reconstruction. In the tissue formation phase, the dECM provides a tissue-specific template guiding recruited host cells and residual endometrial cells to organize into new glands and stroma. Finally, as the scaffold gradually degrades, a well-vascularized, functionally restored endometrium supporting implantation remains. Thus, biomaterials are evolving into active multifunctional platforms that dynamically manage the entire healing process, opening a new frontier for the treatment of Asherman’s.

### Endometriosis

Although strategies for Asherman’s syndrome aim to regenerate a functional endometrium to restore fertility, endometriosis poses a distinct yet related fertility challenge. Unlike Asherman’s syndrome, primarily a structural disorder involving intrauterine scarring, endometriosis is a chronic systemic inflammatory condition characterized by the ectopic growth of endometrial-like tissue. Ectopic tissue precipitates debilitating pelvic pain, inflammation, and adhesion formation that distort pelvic anatomy and impair ovarian and tubal functions. Current interventions, including hormonal suppression and surgical excision of lesions, afford symptomatic relief but not cure and exhibit high recurrence rates after cessation [173]. This observation highlights the necessity for strategies that target the underlying pathophysiology locally. Endometriotic lesions are sustained by a complex microenvironment characterized as proinflammatory, immunosuppressive, and proangiogenic. Biomaterials have emerged as a promising platform to prevent post-surgical complications and deliver targeted therapeutics to endometriotic lesions [174,175].

Post-operative adhesions pose a significant challenge following surgical removal, potentially exacerbating pain and compromising fertility. Various biodegradable anti-adhesion barriers have been developed to address this issue [176]. These materials, frequently formulated as films or in situ forming hydrogels from polymers such as collagen, HA, carboxymethyl cellulose, silk fibroin, and PLGA, are positioned between surgical sites to serve as physical spacers that prevent aberrant tissue connections during healing [177]. HA-based antiadhesion barriers are widely employed to prevent postoperative adhesions following abdominal and uterine procedures. Commercial products such as Hyalobarrier (gel) and Seprafilm (sheet) have been clinically validated to reduce adhesions in gynecologic and colorectal procedures and exhibit favorable safety profiles [178]. Recent advances, including thermosensitive gels such as Mediclore, enable simplified handling and application. These formulations have yielded promising results in clinical studies, especially in pelvic surgery, although efficacy may vary by surgical context.

Advanced strategies have focused on treating the disease by delivering drugs directly to ectopic lesions, thus maximizing local efficacy and minimizing systemic side effects. Injectable hydrogels and nanoparticles represent optimal platforms for this purpose [179]. These systems may be loaded with anti-inflammatory agents, hormonal modulators (e.g., letrozole), or antiangiogenic drugs for suppressing lesion growth and mitigating the inflammatory microenvironment. For example, nanoparticles can enhance the bioavailability of hydrophobic drugs such as curcumin, which exhibits potent anti-inflammatory effects and can be delivered directly to lesion sites [180,181]. Kotb et al. [182] developed chrysin-loaded PLGA nanoparticles (CHR-PLGA-NPs) to address the poor absorption and low bioavailability of free chrysin and evaluated their efficacy in a syngeneic mouse model of endometriosis. The nanoformulation significantly reduced endometriotic lesion implantation by suppressing peritoneal inflammation, inhibiting nuclear factor κB (NF-κB)-mediated inflammatory signaling, reducing angiogenesis and tissue remodeling, and overcoming apoptosis resistance.

Recent studies have explored biological materials for immunomodulation. The peritoneal fluid of patients with endometriosis is enriched in proinflammatory (M1) macrophages, which are essential for lesion survival and growth. Biomaterials can be engineered to release molecules that repolarize these cells toward an anti-inflammatory, pro-resolving (M2) phenotype, restoring immune homeostasis and suppressing disease progression [183,184]. One study determined that direct cell therapy with M1 macrophages significantly reduced lesion number and size [185]. Furthermore, nanovesicles derived from M1 macrophages reprogrammed M2 macrophages, inhibiting key lesion-development processes such as migration, invasion, and angiogenesis [186]. Recently, Wu et al. [187] reported that PLGA nanoparticles coated with bacterial outer membranes effectively suppressed fibrosis and disease progression by converting M2 macrophages to the M1 phenotype (Fig. 4D). By targeting endometriosis’ inflammatory and immunological drivers locally, biomaterial-based therapies offer a more precise and potentially durable approach for fertility preservation in women with this chronic condition.

### Uterine factor infertility: Reconstruction with biomaterials

UFI represents a formidable challenge in reproductive medicine. It arises from congenital uterine absence (e.g., Mayer–Rokitansky–Küster–Hauser syndrome), severe malformations, or acquired damage from extensive surgery, infection, or Asherman syndrome [188]. For women with UFI, the only pathways to motherhood remain uterine transplantation or gestational surrogacy—options fraught with significant medical, ethical, and financial hurdles [189]. Uterine transplantation is a major surgical procedure requiring lifelong immunosuppression to prevent graft rejection, which entails substantial maternal risks [190]. This clinical gap motivated the development of uterine tissue engineering, a regenerative medicine approach to create functional, patient-specific uteri capable of supporting implantation and pregnancy [191].

Uterine tissue engineering is increasingly driven by the quest to reconstruct nature’s complexity through biomaterial scaffolds that emulate the structure and function of the native uterus. Decellularized uterine scaffolds exemplify this approach [170,192] where detergents selectively strip cellular contents while maintaining the delicate architecture of the ECM. The resulting acellular scaffold retains the organ’s native architecture, vascular network, and a rich array of tissue-specific biochemical cues essential for guiding cell attachment, differentiation, and regeneration [193]. Preclinical studies in rodent and rabbit models have demonstrated notable results. Decellularized uterine scaffolds reseeded with recipient endometrial and myometrial cells or stem cells and subsequently transplanted regenerated functional uterine tissue [194]. These bioengineered grafts supported embryo implantation, placentation, and full-term pregnancies, culminating in healthy offspring [195–197]. These outcomes validate the capacity of the native ECM to coordinate complex tissue regeneration.

Moreover, scaffolds fabricated from natural and synthetic polymers offer a highly tunable alternative. Techniques such as electrospinning produce fibrous scaffolds from biocompatible materials, including PLGA, polycaprolactone (PCL), silk fibroin, and collagen [198]. These engineered scaffolds can mimic the distinct biomechanical properties and layered structures of the myometrium and endometrium. They can be loaded with growth factors such as VEGF to promote angiogenesis—a critical step for graft survival and integration [199]. While synthetic scaffolds may not fully capture the biological intricacy of dECMs, their tunable mechanical properties, controlled degradation, and scalable manufacturing render them highly promising for clinical translation. The ultimate goal of uterine tissue engineering is to create autologous regenerative platforms that eliminate dependence on donor organs and immunosuppressive therapy. By integrating advanced biomaterial scaffolds with patient-derived cells, de novo uterus reconstruction is moving from concept toward reality. Although still in preclinical development, this emerging field marks a transformative frontier in reproductive medicine—offering renewed hope that women with UFI may one day bear their own genetic offspring.

## Enabling Technologies and Translational Outlook for Next-Generation Reproductive Biomaterials

**Fig. 5.**
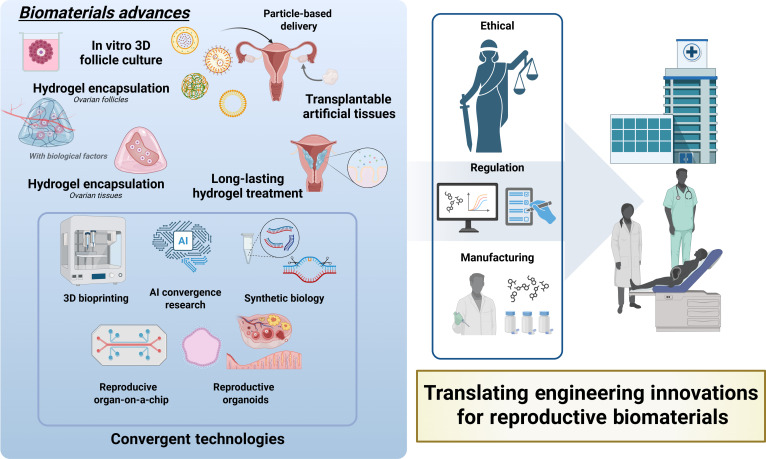
Convergent bioengineering technologies driving translating engineering innovations for reproductive biomaterials beyond biomaterials advances. Schematic illustration of how advanced platforms—including 3D bioprinting, organoid models, microfluidic “organ-on-a-chip” systems, synthetic biology, and artificial intelligence (AI)—synergistically accelerate the design and translation of reproductive biomaterials. 3D bioprinting enables precise reconstruction of ovarian and uterine architectures using tissue-specific bioinks; organoids replicate reproductive microanatomy and function for personalized testing; synthetic biology programs cells and scaffolds for controlled hormone or cytokine release; and AI optimizes material formulations and predicts clinical performance. Together, these convergent technologies bridge fundamental biology and clinical translation, advancing dynamic, patient-specific regenerative platforms for reproductive restoration.

The future of reproductive medicine is shaped not only by these applications but also by the convergence of cutting-edge technologies and by overcoming formidable barriers to clinical translation. The transition from static constructs to dynamic functional systems is achievable only through advanced biofabrication, predictive modeling, and comprehensive understanding of translational pathways. This chapter reviews key enabling technologies that accelerate the development of next-generation biomaterials for reproductive restoration and delineates the significant challenges these innovations encounter on the path from bench to bedside (Fig. 5).

### Convergent technologies for replicating reproductive function: The bioengineering toolkit

Advancements in reproductive tissue engineering increasingly derive from the convergence of complementary technologies instead of isolated breakthroughs, with 3D bioprinting, organoids, synthetic biology, and artificial intelligence (AI) emerging as powerful bioengineering toolkits to replicate reproductive functions. In particular, 3D bioprinting has advanced beyond simple scaffold fabrication to become central in reconstructing the intricate 3D organization of reproductive tissues via precise layer-by-layer deposition of cells and biomaterials [200]. This technique facilitated the fabrication of an artificial ovary characterized by spatially compartmentalized microenvironments that support folliculogenesis and hormone secretion, ultimately restoring fertility in murine models [34]. Central to these developments are bioinks derived from tissue-specific dECMs, which maintain structural fidelity and provide the necessary biochemical milieu for directing cellular behavior and promoting functional tissue regeneration [201].

Concurrent advances in organoid biology have further expanded the toolkit; stem cell-derived organoids accurately recapitulate the microanatomy and function of reproductive tissues such as the ovary and endometrium [202]. When integrated with microfluidic “organ-on-a-chip” systems, these constructs produce dynamic microphysiological environments that simulate complex processes, including the menstrual cycle and disease pathogenesis such as endometriosis and inter-organ communication. Patient-derived organoids from iPSCs or biopsy specimens facilitate the creation of personalized “avatars”, which, when cultured on chips, enable preclinical testing of drugs and biomaterial-based therapies under patient-specific conditions, de-risking clinical trials and advancing precision reproductive medicine [203]. Complementary to these cellular platforms, synthetic biology permits engineering of cells with programmable functions: Applications include smart contraceptive devices that dynamically respond to hormonal fluctuations and bioactive scaffolds that secrete therapeutic molecules on demand [204]. Additionally, AI and machine learning expedite this innovation cycle by analyzing large datasets to predict biomaterial properties, optimize scaffold architecture, and identify novel material formulations, thus reducing reliance on empirical trial-and-error approaches [205]. Indeed, clinically, the AI-related techniques have already settled down enhancing the efficiency and success rates of assisted reproductive technologies like IVF [206–208]. For instance, deep learning models utilizing time-lapse imaging have been developed to automatically identify developmental abnormalities in human embryos [209]. Similarly, the AI approach based on deep neural networks (called “STORK” framework) enables robust assessment of blastocyst quality [210], while other AI models predict embryo viability and implantation potential with high accuracy [211]. Furthermore, AI algorithms are being applied to assess oocyte morphology and predict fertilization outcomes [212]. Despite these rapid clinical advancements, applying these technologies to tissue engineering remains challenging. Currently, the clinicians have struggled to better embryo selection and evaluation, gamete assessment, personalized treatment plans, workflow optimization, and predictive modeling. However, it still remains a challenge to standardize these technologies across different clinical settings and to validate the safety and efficacy of algorithmic decisions through large-scale prospective studies.

Despite rapid technological progress, clinical translation remains challenging because of key biological impediments. Among these obstacles are vascularization and immunomodulation; the survival of engineered constructs larger than several hundred micrometers depends on rapid integration with host vasculature [213], which has led to strategies such as embedding angiogenic growth factors (e.g., VEGF and bFGF), coprinting of endothelial cells to establish prevascularized networks, and engineering microfluidic channels within scaffolds. Currently, immune rejection remains a principal barrier to sustained graft function. Early biomaterial designs sought to achieve immunological silence through inert polymers such as PEG, thereby minimizing host recognition, thereby minimizing host recognition. In contrast, contemporary strategies embrace “immuno-instruction”, wherein scaffolds are engineered to actively modulate host immunity—recruiting and polarizing macrophages toward a pro-regenerative M2 phenotype while locally releasing anti-inflammatory cytokines to foster graft tolerance and seamless tissue integration [214]. Collectively, these convergent technologies exemplify both the promise and complexity inherent in engineering reproductive functions, underscoring the need for coordinated innovation at the nexus of biology, materials science, and clinical translation. This convergence sets the stage for discussions regarding the regulatory and ethical frameworks that will ultimately determine clinical implementation.

### Regulatory, manufacturing, and ethical landscape

Bioengineered products face a complex regulatory pathway and are typically classified as advanced therapeutic medicinal products (ATMPs) [215]. A principal challenge involves developing assays that consistently measure and validate the potency of these complex living products. Accelerated pathways, such as the regenerative medicine advanced therapy (RMAT) designation, enable the development of innovative therapies. Transitioning from bespoke, lab-scale fabrication to robust, scalable, and reproducible manufacturing processes under good manufacturing practices (GMPs) is essential for advancing to clinical trials and commercialization. A rigorous assessment of long-term safety, including the potential tumorigenicity of implanted cells and the durability of regenerated tissues, is required [216]. Moreover, engineering human reproductive tissues, particularly those involving germ cells or the creation of artificial uteri, must be accompanied by comprehensive ethical evaluations [217].

Regulatory complexities prevail across various biomaterial platforms in the reproductive field. Commercial cryopreservation products, such as vitrification kits from Cryotop (Kitazato) and FertiVit (FertiPro), have achieved clinical adoption; however, they remain subject to ongoing GMP and sterility compliance updates and long-term offspring monitoring requirements [218,219]. In contrast, hydrogel-based artificial ovaries and in vitro follicle maturation systems remain confined to preclinical studies, as regulators require multigenerational safety data and proof of genetic stability before human trial approval. Similarly, nanoparticle-based hormone delivery systems, including PLGA–E2 depots, demonstrate promise but face investigational new drug (IND)-related hurdles owing to systemic toxicity risks, in sharp contrast to conventional, regulator-approved hormone drugs such as Gonal-F or Lupron Depot [220]. Tissue-engineered and 3D-bioprinted endometrial or ovarian scaffolds face greater challenges because their combination of living cells and biomaterials renders classification ambiguous, often necessitating case-by-case evaluation as ATMPs or human cells, tissues, or cellular or tissue-based products (HCT/Ps) [221,222]. While simpler acellular scaffolds for adhesion prevention are already in or near clinical use, complex bioengineered organs such as artificial ovaries remain largely at the preclinical stage using murine or ovine models, likely requiring 5 to 10 years for clinical translation due to the necessity for rigorous safety studies under GMP. These examples illustrate that, unlike relatively mature cryopreservation platforms, most next-generation reproductive biomaterials are hindered by the absence of harmonized global regulations, standardized manufacturing, and rigorous long-term outcome studies.

## Conclusions and Future Perspectives

Biomaterials have evolved from simple structural scaffolds to central components in regenerative reproductive medicine. 3D hydrogel-based follicle culture systems have demonstrated substantial success in recapitulating the ovarian microenvironment, while bioprosthetic ovaries generated through 3D printing have restored endocrine function and produced live offspring in animal models. Parallel to these material innovations, the integration of AI is driving a shift toward precision reproductive medicine. AI-driven deep learning tools are already transforming clinical workflows through automated embryo selection and gamete quality assessment. The convergence of these technologies with biomaterial engineering is anticipated to enable patient-specific therapeutic design, wherein AI can guide scaffold composition, predict clinical outcomes, and optimize bioink formulations and degradation profiles using datasets derived from organ-on-a-chip platforms. Emerging strategies employing biomaterials and 4D bioprinting further enable the development of constructs capable of responding to physiological cues, thereby expanding their therapeutic scope beyond fertility preservation to include conditions such as Asherman syndrome and UFI.

Despite these promising developments, key translational barriers remain. Rapid vascularization of large-volume constructs, modulation of host immune responses, and the establishment of standardized GMP-grade manufacturing and regulatory pathways are pressing challenges that require interdisciplinary solutions. Ensuring responsible innovation will also require a robust governance framework aligned with international guidelines, such as those issued by the International Society for Stem Cell Research (ISSCR), and active public involvement to address the ethical implications associated with emerging technologies, including artificial gestational platforms and germline engineering. Although this review primarily focuses on female reproductive applications, the discussed bioengineering principles—particularly hydrogel-based niche reconstruction and dECM platforms—are also relevant to male reproductive restoration. These methodologies are being translated to male reproductive research, particularly in the development of testicular organoids and platforms for spermatogonial stem cell transplantation. Together, the emerging applications suggest that shared bioengineering principles are poised to progressively integrate advancements in both male and female reproductive systems, paving the way for future innovations in reproductive medicine.

## Data Availability

This review did not generate new data. All data and information included are from previously published sources cited in the manuscript.
